# Amelioration of cognitive impairments in APPswe/PS1dE9 mice is associated with metabolites alteration induced by total salvianolic acid

**DOI:** 10.1371/journal.pone.0174763

**Published:** 2017-03-30

**Authors:** Li Shen, Bing Han, Yuan Geng, Jinhua Wang, Zhengmin Wang, Mingwei Wang

**Affiliations:** 1 Clinical Laboratory, The First Hospital of Hebei Medical University, Shijiazhuang, Hebei Province, China; 2 Department of Neurology, The First Hospital of Hebei Medical University, Shijiazhuang, Hebei Province, China; 3 Brain Aging and Cognitive Neuroscience Laboratory of Hebei Province, The First Hospital of Hebei Medical University, Shijiazhuang, Hebei Province, China; Nathan S Kline Institute, UNITED STATES

## Abstract

**Purpose:**

Total salvianolic acid (TSA) is extracted from salvia miltiorrhiza; however, to date, there has been limited characterization of its effects on metabolites in Alzheimer’s disease model-APPswe/PS1dE9 mice. The main objective of this study was to investigate the metabolic changes in 7-month-old APPswe/PS1dE9 mice treated with TSA, which protects against learning and memory impairment.

**Methods:**

APPswe/PS1dE9 mice were treated with TSA (30 mg/kg·d and 60 mg/kg·d, i.p.) and saline (i.p.) daily from 3.5 months old for 14 weeks; saline-treated (i.p.) WT mice were included as the controls. The effects of TSA on learning and memory were assessed by a series of behavioral tests, including the NOR, MWM and step-through tasks. The FBG and plasma lipid levels were subsequently assessed using the GOPOD and enzymatic color methods, respectively. Finally, the concentrations of Aβ42, Aβ40 and metabolites in the hippocampus of the mice were detected via ELISA and GC-TOF-MS, respectively.

**Results:**

At 7 months of age, the APPswe/PS1dE9 mice treated with TSA exhibited an improvement in the preference index (PI) one hour after the acquisition phase in the NOR and the preservation of spatial learning and memory in the MWM. Treatment with TSA substantially decreased the LDL-C level, and 60 mg/kg TSA decreased the CHOL level compared with the plasma level of the APPswe/PS1dE9 group. The Aβ42 and Aβ40 levels in the hippocampus were decreased in the TSA-treated group compared with the saline-treated APPswe/PS1dE9 group. The regulation of metabolic pathways relevant to TSA predominantly included carbohydrate metabolism, such as sorbitol, glucose-6-phosphate, sucrose-6-phosphate and galactose, vitamin metabolism involved in cholecalciferol and ascorbate in the hippocampus.

**Conclusions:**

TSA induced a remarkable amelioration of learning and memory impairments in APPswe/PS1dE9 mice through the regulation of Aβ42, Aβ40, carbohydrate and vitamin metabolites in the hippocampus and LDL-C and CHOL in the plasma.

## Introduction

Alzheimer’s disease, a widespread type of dementia in elderly individuals, is an age-associated neurodegenerative disease characterized by ongoing episodic memory impairment and progressive cognitive deficiencies [1]. The symptoms are severe and affect work, hobbies and social life. To date, there are approximately 50 million senior patients with AD, and in China, which has a population in excess of one billion, patients with AD comprise more than 5.1 million (World Health Organization, 2012). More than one-half of elders older than 85 years of age experience the pain of AD worldwide [2]. The aging situation is currently grim; thus, research on the preventive medicine is meaningful and extremely urgent for patients with AD to escape from the disorder or ameliorate the scourge of dementia syndromes.

The major pathological hallmarks of AD, which are mainly localized in the cortex and the hippocampus, comprise extracellular Aβ-rich SPs, NFTs and the loss of synapses [3], and these pathological lesions are linked to cognitive decline [4]. SPs are one of the most important characteristics of AD; as its core constituents, Aβ40 and Aβ42 are pivotal to AD pathogenesis [5] and have been proposed as the driving force of the disease process in the “amyloid cascade hypothesis”, which lead to neuronal degeneration [6] and in which Aβ42 is considered the crucial pathogenic driving force [7].

APPswe/PS1dE9 gene mutations identified as the Swedish-mutated amyloid precursor protein gene combined with the exon-9-deleted presenilin 1 gene recapitulate the onset and progression of early-onset familial AD in humans [8]. As a useful tool for research on AD, APPswe/PS1dE9 transgenic mice reproduce the aging-dependent cognitive decline present in the gradual process of AD [9]. Moreover, substantial quantities of Aβ42 and the ratio of Aβ42 to Aβ40 have been identified in the brain in previous reports [10], which is similar to the findings for patients with AD [11].

There may be complex latent biomarkers accompanied by pathogenic changes related to AD; thus, metabolomics plays an outstanding role in biomarker discovery and identification during the progressive course of AD. GC-TOF-MS in conjunction with MSA, which includes its own advantages of high sensitivity, peak resolution, reproducibility and high throughput, have been used for the analysis of metabolite perturbations associated with the disease in serum and other samples [12, 13]; however, there have been fewer reports on early-onset biomarkers associated with AD and the corresponding preventive medication [14]. In recent research, the metabolic profiles of APPswe/PS1dE9 mice in different brain regions have been documented [15]. Based on these metabolites, we assessed the drug therapeutic effect for AD, and we will also discover novel biomarkers that impact the progressive process of AD for drug design. However, to our knowledge, it has remained unclear to date which metabolites in the hippocampus were altered in APPswe/PS1dE9 double transgenic mice aged 7 months after approximately 3 months of TSA treatment. Despite the efforts of researchers to perform AD treatments for 12 years, unfortunate results exhibited terrible success rates of less than 3% for the drugs [16]. Freeze-dried TSA sterile power, a type of polyphenolic compound extracted from the water-soluble effective parts in salvia miltiorrhiza bunge, comprises a type of new traditional Chinese medicine, which is transformed from traditional oral administration to intravenous infusion [17]. It has several oxidative and neuroprotective effects on transient ischemia in a previous study [18]. To date, there have been few characterizations of metabolites in the blood or hippocampus following TSA treatment in APPswe/PS1dE9 mice. We aimed to clarify through our research whether TSA could induce neuroprotective effects on learning and memory. Furthermore, we investigated which metabolites were regulated in the hippocampus and plasma of TSA-treated APPswe/PS1dE9 transgenic mice.

## Materials and methods

### Animals and administration

All animal-associated experiments have approval from the Committee on the Ethics of Animal Experiments of the First Hospital of Hebei Medical University (Permit Number: 20150006) and the experiments were performed according to the Regulations for the Administration of Affairs Concerning Experimental Animals, which was approved by the State Council of the People’s Republic of China on October 31, 1988, and promulgated by Decree No. 2 of the State Science and Technology Commission on November 14, 1988. Expressing chimerical APP with K595N and M596L, the Swedish double mutation and deleted-exon-9 presenilin 1 mutation, male APPswe/PS1dE mice were obtained from Nanjing University Institute of Animal Models, license for experimental animals: SCXK (苏) 2010–0001.

The WT mice comprised male C57BL6/J mice, which were age-matched with the same background of the APPswe/PS1dE9 transgenic mice. We utilized twenty male mice from 3.5 months old for our research. The housing conditions were as follows: light from 07:00 to 19:00, temperature range 22± 2°C, and humidity range 55±10%. The mice obtained fresh water and food ad libitum.

### Experimental groups and process

There were four groups in our research. The APPswe/PS1dE9 transgenic group and WT control group were treated with an equal volume of vehicle (NS, i.p.). The TSA-treated groups included APPswe/PS1dE9 mice treated with total salvianolic acid (Tianjin tasly co., LTD, Tianjin, China) at doses of 30 mg/kg·d and 60 mg/kg·d (TSA, i.p.). The acute and chronic toxicity experiments had been performed and certificated by Institute of Materia Medica in Chinese Academy of Medical. The lethal dose 50 (i.p.) of mice was at 1994.34 mg·kg^-1^ and there was no obvious toxic injury of pathology below 200 mg/kg dose on 21 organs of mice including brains. In addition, the experiments of pharmacokinetics had been performed by Tianjin University of TCM and Tianjin International Joint Academy of Biomedicine recently. There were 5 male mice per group and 20 mice were used in the experiment. We measured the body weight every week in the course of the experiment to determine the total quantity of TSA for intraperitoneal injection per mouse.

Daily treatment was administered to the mice from the age of 3.5 months and continuing for 14 weeks. After consecutive treatment, behavioral research experiments on the mice were performed for 10 days. During the period of the behavioral experiments, no treatment was administered to the mice. On the 10^th^ day of the behavioral experiments, fasting was initiated and maintained for 16 hours. The next morning, from the tip of the mouse tail, every blood sample was collected for the determination of the glucose level via the method of Mut.Q-GDH using an Accu-Chek Performa glucose meter (Roche Diagnostic GmbH, Mannheim, Germany). The mice subsequently received isoflurane anesthesia, and blood samples were collected from the eyeballs of the mice. The blood was anti-coagulated using EDTA-2K and centrifuged at 19*100 (× g) for 8 minutes at room temperature; the plasma was drawn from the centrifuge tube and maintained at -80°C for lipid detection. The hippocampal tissue was rapidly separated on ice and stored at -80°C for subsequent metabolite analysis.

### Novel Object Recognition task (NOR)

Taking advantage of the innate exploratory behavior of animals, the NOR task was the first behavior test to assess memory after treatment. The protocol was adjusted according to the previous method of the NOR [19]. Two days were required to perform the NOR task, which assessed the innate curiosity or exploratory instinct of the mice. The experimental temperature was maintained at 27±1°C. The objects to be discriminated with different shapes and colors were made of wood and were pasted on the bottom of an open field in a white square plastic chamber (50 × 50 × 45 cm). On the first day of the NOR task, the mice were placed in the testing arena for 30 minutes and allowed to habituate to the surroundings in a bright room. We offered each mouse two indistinguishable objects near two opposite walls of the open field, and the mice were trained to freely explore for ten minutes (acquisition task). During the training period, the mice were placed at the midpoint of the open field without shavings. Two indistinguishable objects in terms of shape and color (pink, octahedral wooden block) were situated in the north and south quadrants, spaced an equidistant 15 cm from the walls of the open field (position A and position B). The exploring criterion was that the mice sniffed or touched the blocks, and the distance was less than 2 cm away from the block when the mice orientated toward the blocks.

During the trial interval in which the mice were back in the cage, we cleaned the housing cage with 70% ethanol to remove the olfactory cues. After one hour of retention, we replaced one of the objects with a novel object of similar size (green, triangle cylinder wooden block) and recorded the exploring time spent touching the two objects. The preference index (PI) represents the novel object exploration percentage, which comprises the time touching the novel object divided by the total time exploring the two objects. After 24 hours, the triangle cylinder wooden block was placed with another novel object (red, wooden cuboid block), and the exploration time spent touching the identical and novel object was assessed within 5 min; the PI was measured again 24 hours after the first exploration. The experimental procedure, including the frequency with which the mice touched the object and the time that the mice explored two objects, was tracked by a UVC 400 Video Device and analyzed with the video tracking system of ANY-maze (Stoelting Co., Ltd, Wood Dale, Illinois, USA).

### Morris Water Maze (MWM)

The MWM comprised the second behavioral test used to assess learning and spatial memory after treatment according to the method of Morris [20]. Whole tests were divided into two trials, including the spatial navigation trial (5 days) and the probe trial (1 day). During spatial navigation trial, we placed various prominent visual cues above the wall from four planned four cardinal points (N, S, E, W), and the mice were individually placed in the water; they faced a wall of a 120-cm-diameter circular cistern filled with opaque water (22±2°C) containing white food dye at a depth of 41 cm. The spatial navigation trial ended when the mice climbed onto the platform (8 cm in diameter, submerged 1 cm under water, located in the middle of the third quadrant) and remained on it for 3 s; the latencies were recorded. The latencies were recorded at 60 s if the mice could not find the platform within 1 minute. We led the mice onto the platform and trained them to remain there for 10 s. The parameter was averaged in each training session for each mouse. Four acquisition trials were performed every day during five consecutive days with a five-minute inter-trial interval. The arrangement of the cardinal point in the four trials was conducted as previously reported [21]. On the sixth day, we removed the platform and performed the probe trial for 60 s to determine whether the mice could remember the previous location of the platform. The latencies searching for the hidden platform, time ratio (percentages of time spent in the target quadrant where the platform had been previously located) and the frequency crossing the previous target platform were recorded and analyzed using Super Maze Animal behavior analysis system and image analysis software (Shanghai Xin Ruan Information technology co., LTD, Shanghai, China).

### Step-Through Passive Avoidance task (STPA)

Following the MWM, we evaluated the memory capability of the mice in the STPA task according to the literature [22]. The apparatus consisted of an illuminated compartment that placed a lamp 1 meter above and an identical non-illuminated compartment divided by an automatic lifting gate. Stainless steel rods were placed on the floor of both compartments 0.5 cm apart. There were three trials of the step-through task: the habituated trial, training trial and retention trial. During the habituated trial period, each mouse was maintained in the illuminated chamber for 5 minutes to adapt to the chamber. In the training trial, we initially chambered the mice in the illuminated room. Once the mice crossed the threshold of the dark compartment, the automatic lifting gate closed, and two seconds later, an electrical shock (0.3 mA, 10 s) was delivered through stainless steel rods. After the foot shock, we returned the mice to their cages individually. We performed the retention trial 24 hours after the training trial. The latencies were analyzed using Super Passive Avoidance Video analysis system and image analysis software (Shanghai Xin Ruan Information technology co., LTD, Shanghai, China); we defined them as the time spent entering the dark compartment for the training trial and the retention trial. The latencies of both experiments were recorded for up to 180 s.

### Determination of blood glucose and plasma lipid

Following an overnight fast (16 hours), blood glucose was detected using the Mut.Q-GDH method according to the instructions. The lipids assessed included CHOL, TG, HDL and LDL. The quantitative determination of the four lipids in the plasma was conducted by the enzymatic color test on Beckman Coulter AU5800 biochemical analyzers (Beckman Coulter, Inc., Brea, California, USA) [23].

### Cerebral hippocampus tissue preparation for Aβ40 and Aβ42 detection

Ten milligrams of hippocampal tissue was homogenized in an Eppendorf tube that contained a 50-μl mixture (pH = 8.0) of 5 M guanidine HCl and 50 mM Tris HCl (Invitrogen Corporation, Camarillo, California, USA) and was thoroughly ground using a hand-held micro-homogenizer with a sterilized tip (Fisher Scientific, Vantaa, Finland). The homogenates were maintained at RT for 4 hours and stored at -80°C prior to the detection of Aβ40 and Aβ42. Cold BSAT-DPBS (Pierce Manufacturing, Inc., Appleton, Wisconsin, USA) that contained protease inhibitor cocktail (EMD Millipore Corporation, San Diego, California, USA) were used as diluents. The hippocampus homogenates were centrifuged (16,000 x g) for 20 min at 4°C. Aβ40 and Aβ42 in the supernatants were assayed according to the manufacturers’ protocols. The dilution factors of Aβ42 and Aβ40 detection were 500 and 50, respectively. The supernatants were carefully decanted and stored on ice until the detection of Aβ42 and Aβ40.

### Assay method of Aβ40 and Aβ42 by ELISA

The Aβ42 and Aβ40 levels in 100-μl samples and the corresponding standards were detected using ELISA with Aβ42 and Aβ40 ELISA kits (Invitrogen Corporation, Camarillo, California, USA). At 450 nm, the absorbance was read by a Multiskan Spectrum Microplate Spectrophotometer (Thermo Fisher Scientific Oy Ratastie 2, Vantaa, Finland), which was used to quantitate the Ms Aβ 40 or Ms Aβ 42 concentrations according to the standard curve plotted. The Aβ42 and Aβ40 levels were subsequently standardized and expressed as pg/mg (Aβ42 or Aβ40 mass/hippocampus tissue weight).

### Cerebral hippocampus tissue preparation for metabolite detection by GC-MS

Extraction of metabolites: Each hippocampus sample was put into 2-ml EP tubes and extracted with 0.1-ml mixtures that contained methanol and chloroform (volume ratio = 3:1) with L-2-chloro phenylalanine (10 μl) as the internal standard (Shanghai hengbai technology co., LTD, Shanghai, China). The mixtures had been homogenized for 3 min at 65 Hz in a JXFSTPRP-24 ball mill (Shanghai Jingxin Industry Development Co. LTD, Shanghai, China); at 4°C, they were centrifuged (12,000 rpm) for 15 min. We transferred the supernatant (80 μl) into a GC-MS glass vial (2 ml) for derivatization. In addition, an equal volume of 2 μl (based on the number of samples) was obtained from each sample and placed into a GC-MS glass vial (2 ml) to assess the systematic stability during the detection process [24].

Derivatization of metabolites [25]: The extracts were dried in a vacuum concentrator without heating for approximately 3 hours at 37°C and redissolved in 20 μl of methoxylamine hydrochloride, which was dissolved in pyridine (20 mg/ml) for the protection of carbonyl groups. Following vortexing, the samples were incubated at 80°C for 20 min. Then, 30μl of BSTFA (Regis Technologies Inc., Morton Grove, USA) that contained 1% (v/v) TMCS was added to every sample; the samples were then sealed and incubated at 70°C for 1 hour. Five microliters of FAMEs (standard mixture of fatty acid methyl esters, C8-C16:1 mg/ml; C18-C24:0.5 mg/ml in chloroform) was added to every sample until cooling to RT. The mixture was prepared for subsequent GC-MS analysis.

### Metabolic detection by GC-TOF-MS

The GC-TOF-MS analysis was performed using an Agilent 7890 gas chromatograph system (Agilent Technologies Inc., Santa Clara, California, USA) coupled with a Pegasus HT time-of-flight mass spectrometer (LECO Corp., St. Joseph, MI, USA) that included an Rxi-5Sil MS column (Restek, Bellefonte, USA), which was 30 m in length and 0.25 mm in inner diameter with a film thickness of 0.25 μm. We injected one microliter of sample in splitless mode. The carrier gas was helium with a 3 ml/min front inlet purge flow and a 2 ml/min gas flow rate through the column. The original temperature was maintained at 50°C for 1 min; the temperature was then increased to 330°C at a 10°C/min rate and maintained for 5 min. The ion source, transfer line and injection temperature were at 250°C, 280°C and 280°C, respectively. We performed the ionization with a voltage of -70 eV in the mode of electron impact during the mass spectrometry analysis process. The mass spectrometry data were acquired in the full-scan mode in the m/z range of 85–600 at a rate of 20 spectra per second after a solvent delay of 366 s.

### Metabolite processing data

LECO Chroma TOF4.3X software and LECO-Fiehn Rtx5 database (Leco Corp., St. Joseph, MI, USA) were used for the raw data acquisition and processing, such as the raw peak exacting, data baseline filtering and calibration, peak alignment and normalization, deconvolution analysis, peak identification based on feature detection and integration of the peak area from the total ion chromatography (TIC). We took advantage of the RI (retention time index) method in the identification of the peak and the tolerance of the RI was 5000 [26]. These original data included the retention times, retention indices, spectral similarity values and spectral information for identification. In addition to the retention time, the characteristic of each metabolite, the definitive structural identification of each substance was provided by its own mass pattern, which provided excellent specificity. The alignment of the data was performed according to the retention time and spectra.

The SIMCA 14.0 software package (Umetrics, Umea, Sweden) was used for the alignment and area normalization of the compounds for further analyses. To better analyze the downstream data, the data should be filtered with the purpose of removing the noise data using the method of the interquartile range. The data were normalized according to the peak area normalization method [27]. At the end of the data processing, the data were submitted to the subsequent multivariate analysis.

### Multivariate analysis: Principal Component Analysis (PCA) and Orthogonal Projections to Latent Structures-Discriminate Analysis (OPLS-DA)

The three-dimensional multivariate analysis data, which included the peak number, sample name, and normalized peak area, were subjected to a multivariate analysis to perform PCA at the same time to illustrate the distribution of the data with the SIMCA 14.0 software package (Umetrics, Umea, Sweden). Subsequently, as an effective approach to sift metabolites, the scores plot of OPLS-DA was achieved to detect differences and filter more variations between the groups [28]. The quality of the models was assessed by the R^2^ and Q^2^ values in OPLS-DA, which represented the variance and the predictability derived from the models. After assessing in OPLS-DA, two hundred permutations were performed, and the resulting R^2^ and Q^2^ values were plotted to further assess the model validity. We ascertained the metabolites based on the data of the LECO/Fiehn Metabolomics Library.

#### Metabolite identification

A loading scatter plot was initially applied in OPLS-DA. As a result of filtering out the irrelevant orthogonal signal, it was more reliable to obtain metabolites with differences. The first principal component of Variable Importance in the Projection (VIP) was subsequently obtained to refine the analysis, and T test with Bonferroni correction was used to assess the difference via the SIMCA 14.0 software package (Umetrics AB, Umea, Sweden). A VIP that exceeded 1 and a *p*-value less than 0.05 indicated a changed biomarker [26, 28]. The qualitative method of the metabolites matched the substance to a self-building standard substance database and the LECO-Fiehn Rtx5 database (Leco Corp., St. Joseph, MI, USA), which could execute automatic peak identification and the fidelity solution of the convolution, considering that metabolites with a similarity index (SI) greater than 70% represented reliable potential biomarkers [27].

### Statistical analysis

The data are presented as the mean±SEM, and “*n*” denotes the amount of mice per group. The statistical methods used to analyze the navigation data of the MWM, such as the escape latency, included repeated measure ANOVA, and one-way ANOVA was used to analyze other measurement data, including the probe data of the MWM, with the LSD-t test for comparisons between the groups. SPSS 21.0 software (IBM Corporation, Armonk, NY, USA) was used for the statistical analysis, and the SIMCA 14.0 software package (Umetrics AB, Umea, Sweden) was used for the multivariate analysis [26, 29]. The differences were considered significant statistically when the *p*-value was ≤0.05.

## Results

### TSA (30 mg/kg BW/day, i.p.) rescued non-spatial cognitive decline in the NOR test

**Fig 1:**
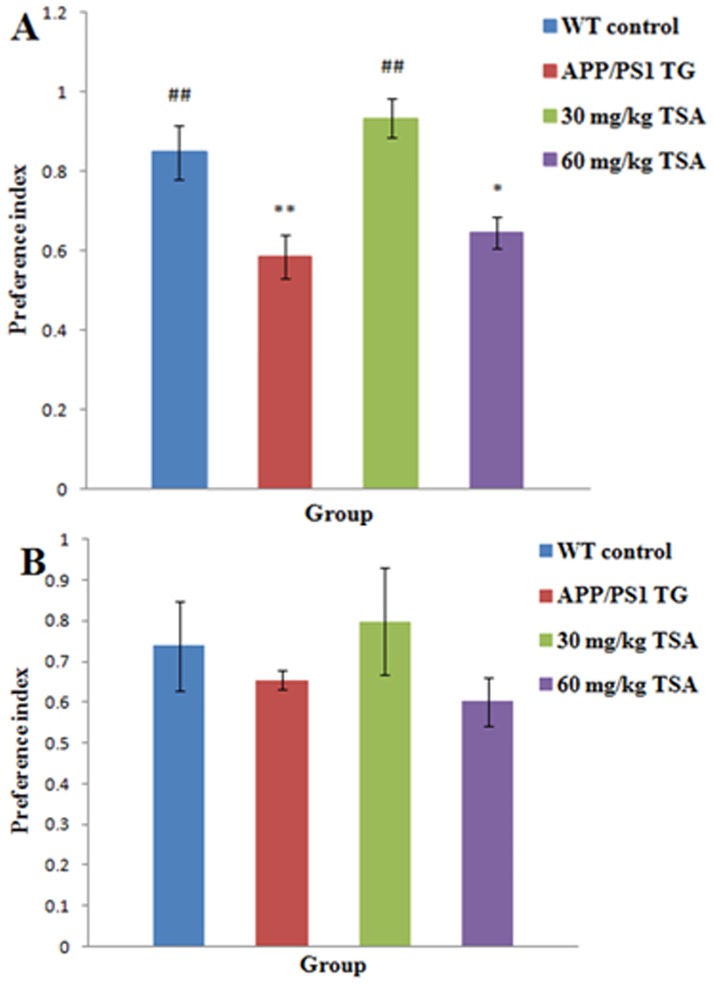
A dose of 30 mg/kg TSA by intraperitoneal injection rescued the non-spatial cognitive impairment of APPswe/PS1dE9 transgenic mice aged 7 months. APPswe/PS1dE9 transgenic mice aged 7 months distinguished the novel object worse than the WT control mice, which indicated a poorer performance in the NOR. The deficits were alleviated by the treatment with 30 mg/kg TSA for 3 months from 3.5 months old. The preference index of the cognitive performance in the NOR of the four groups was expressed as the proportion of time spent exploring the novel object (time spent exploring the novel object/total time of exploration during the testing phase). (A) Preference index after 1-hour retention interval. (B) Preference index after 24-hour retention interval. WT, wild type; TG, transgenic; TSA, total salvianolic acid; *n* = 20, error bars represent SEM. *p<0.05, **p<0.01 versus WT control group; #*p*<0.05, ##*p*<0.01 versus vehicle-treated APPswe/PS1dE9 transgenic group.

To determine whether treatment with TSA improved cognitive impairment, we conducted a behavioral experiment associated with the non-spatial cognitive memory of the mice in the NOR task 1 hour and 24 hours after the training task. As indicated in Fig 1A (raw data are presented in S1 Table), the preference index (PI) of the WT control mice was increased compared with that of the APP/PS1 mice one hour after the acquisition memory phase (*p* = 0.003), which indicated that the mice in the WT group exhibited an increased tendency to explore the new object compared with the APP/PS1 mice. Intraperitoneal injection of TSA at a dose of 30 mg/kg obviously improved the PI of the APP/PS1 mice (*p* = 0.000), which was comparable to that of the WT control mice (*p* = 0.282) and indicated 30 mg/kg TSA (i.p.) rescued the dysfunction of short-term memory in the mice with AD. However, 60 mg/kg TSA did not exhibit this function of improving the PI though the difference was limited (*p* = 0.017) between 60 mg/kg TSA-treated and WT control group. In addition, the results demonstrated that it had no impact on the long-term memory of the APP/PS1 mice 24 hours after the familiarization period [*F* (3, 20) = 0.908, *p* = 0.459] as indicated in Fig 1B (raw data are presented in S1 Table); however, it had an effect on the short-term memory of the APP/PS1 mice.

### TSA (30 mg/kg and 60 mg/kg BW/day, i.p.) improved spatial cognitive impairment in the MWM experiment

**Fig 2:**
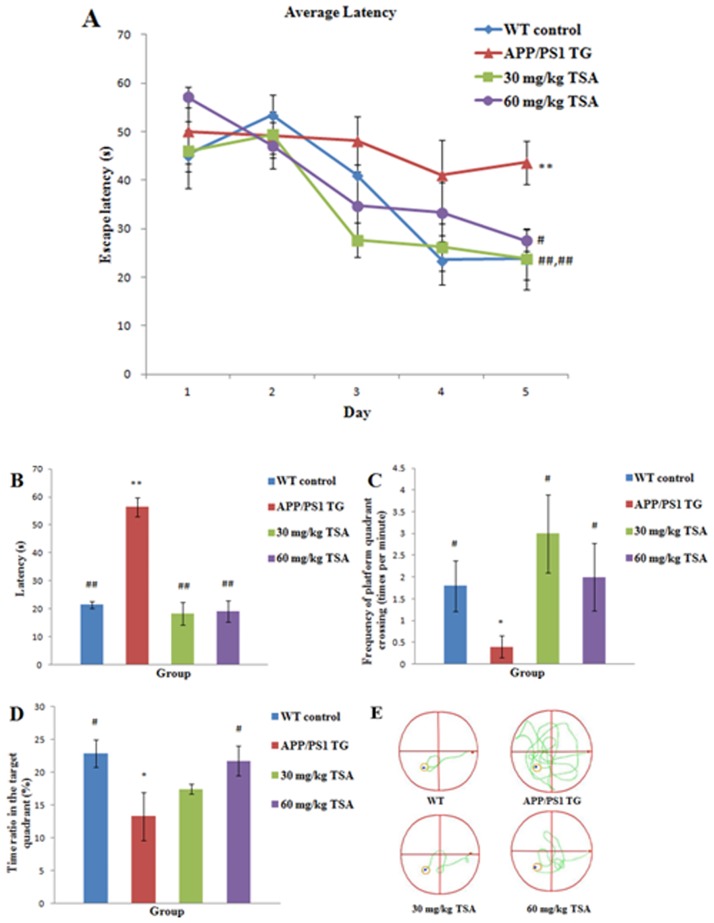
Treatment with TSA protected against spatial learning and memory impairment in APPswe/PS1dE9 transgenic mice aged 7 months in the task of the Morris water maze. In the spatial navigation trials, each point represented the mean escape latency to find a hidden platform on each day of consecutive training (A). The latencies in the probe trial (B), the frequency crossing the target platform (C) and the time ratio spent in the target quadrant (D) are also presented in the probe trial. Representative swimming paths (start, end) of the four groups travelling on the fifth day of the spatial navigation trials (E) are indicated. Data are presented as the mean±SEM, *n* = 20.*p<0.05, **p<0.01 versus WT control group; #*p*<0.05, ##*p*<0.01 versus vehicle-treated APPswe/PS1dE9 transgenic group.

We conducted MWM which was the second behavioral experiment as indicated in Fig 2 associated with the spatial cognitive memory of the mice following the NOR. As indicated in Fig 2A (raw data are presented in S2 Table), in the spatial navigation trials, there was no significant difference in the escape latency of the four groups during the former four days (*p* = 0.410; *p* = 0.459; *p* = 0.105; *p* = 0.190). Nevertheless, on the fifth day of the spatial navigation trials, the escape latency of the APPswe/PS1dE9 transgenic mice was prolonged appreciably compared with the WT control mice (*p* = 0.007). At this time, both doses of TSA (i.p.) reversed the impairments (30 mg/kg TSA, *p* = 0.007; 60 mg/kg TSA, *p* = 0.024) and improved the swimming strategy with which the mice tracked down the platform as indicated in Fig 2E. These findings suggested that the intraperitoneal injection of TSA significantly improved the learning capability of the mice. As indicated in Fig 2B (raw data are presented in S3 Table), there was a difference in the latency among the four groups [*F* (3, 20) = 30.74, *p* = 0.000] in the probe trial. The latency of the APP/PS1 double transgenic mice aged 7 months was substantially prolonged compared with that of the WT control mice (*p* = 0.000). It is gratifying that there was no significant difference in the latency between the TSA-treated groups and the WT control group in the probe trial (30 mg/kg TSA, *p* = 0.511; 60 mg/kg TSA, *p* = 0.623). The frequency of passing across the location of the target platform was different among the four groups in Fig 2C (*x*^2^ = 9.231, *p* = 0.026) (raw data are presented in S3 Table), and it decreased in the APPswe/PS1dE9 group versus the WT control group (*p* = 0.032). Treatment with 30 mg/kg and 60 mg/kg TSA multiplied the frequency crossing in the removal target platform relative to its positive control group (30 mg/kg TSA, *p* = 0.013; 60 mg/kg TSA, *p* = 0.032). In the APPswe/PS1dE9 group, the time ratio for sailing inside the target quadrant decreased compared with the WT control group as indicated in Fig 2D (*p* = 0.013) (raw data are presented in S3 Table). The time ratio substantially increased after approximately nearly 3 months of intraperitoneal injections with a high dose of TSA compared with the positive control group (*p* = 0.026). These findings suggested that TSA (i.p.) improved the spatial cognition of 7-month-old APPswe/PS1dE9 mice.

### Fear memory was unaffected in the step-through passive avoidance task in 7-month-old APPswe/PS1dE9 mice

**Fig 3:**
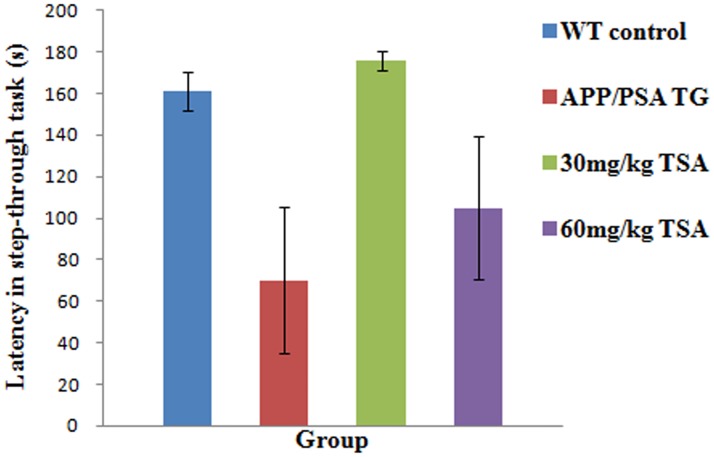
Comparison of retention latency 24 hours after foot shock. There was no significant difference among the WT control, APPswe/PS1dE9 transgenic, or 30 mg/kg or 60 mg/kg TSA-treated groups. Data are presented as the mean±SEM, *n* = 20.

The step-through test is based on conditioned fear behavior. The retention latency exhibited on a degressive tendency in the APPswe/PS1dE9 group; nevertheless, it was not significantly different among the four experimental groups 24 hours after the acquisition test illustrated in Fig 3 (*x*^2^ = 4.417, *p* = 0.220) (raw data are presented in S4 Table). This finding suggested that 7-month-old APP/PS1 transgenic mice were not substantially influenced by fear memory or impaired in terms of long-term memory.

### Determination of Aβ42 and Aβ40 levels in the hippocampus homogenates of mice

**Fig 4:**
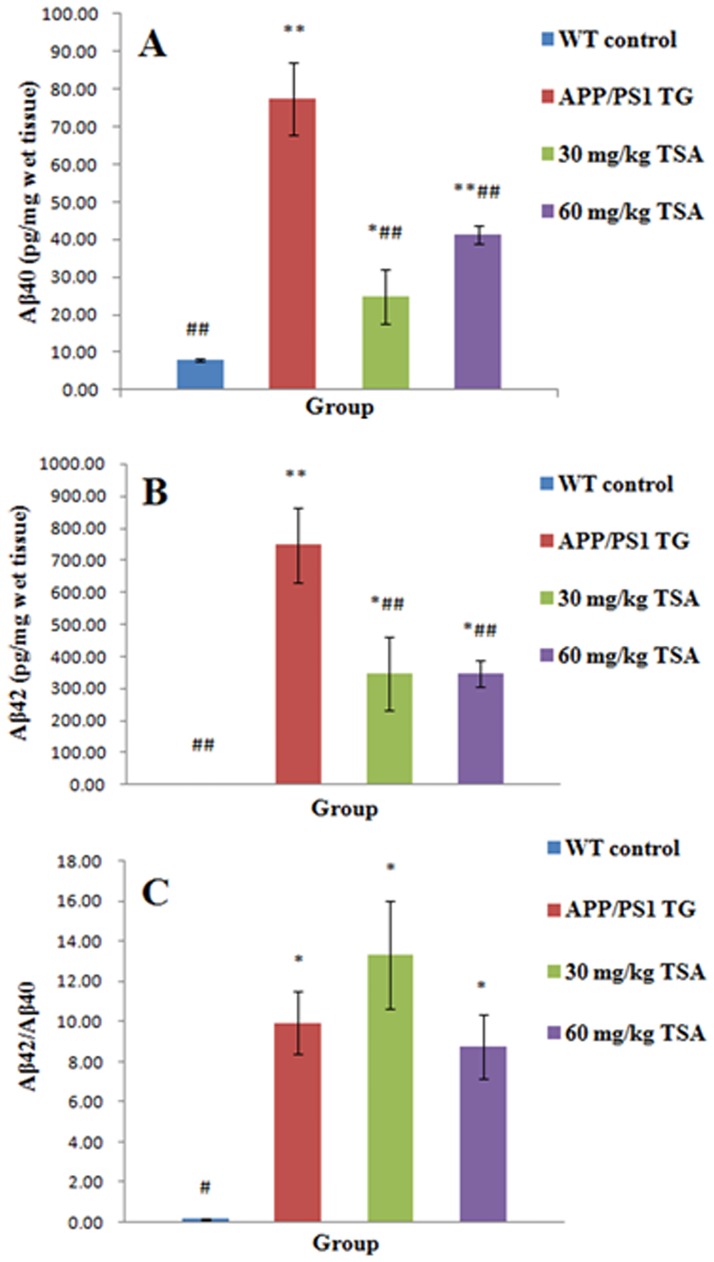
Effect of treatment with TSA on Aβ42 and Aβ40 levels in APPswe/PS1dE9 transgenic mice. The Aβ42 and Aβ40 levels in the hippocampus of the WT control mice, vehicle-treated APPswe/PS1dE9 transgenic mice, and the APPswe/PS1dE9 transgenic mice treated with 30 mg/kg or 60mg/kg TSA were measured by ELISA. (A) Aβ40 was significantly decreased in two TSA-treated groups. (B) Aβ42 was notably decreased after treatment with 30 mg/kg and 60 mg/kg TSA. (C) After treatment with TSA, the ratio of Aβ42 to Aβ40 was not significantly altered. Data are presented as the mean±SEM, *n* = 20.*p<0.05, **p<0.01 versus WT control group; #*p*<0.05, ##*p*<0.01 versus vehicle-treated APPswe/PS1dE9 transgenic group.

As a result of the mutations of the APPswe gene and PS1dE9 gene, this type of transgenic mice aged 7 months accumulated more Aβ40 and Aβ42 in the hippocampus compared with the WT control group, as indicated in Fig 4A and 4B (Aβ40 *vs*. APPswe/PS1dE9 group *p* = 0.000; Aβ42 *vs*. APPswe/PS1dE9 group *p* = 0.000) (raw data are presented in S5 Table). Intraperitoneal injection of 30 mg/kg and 60 mg/kg TSA reduced not only the easily dissolved Aβ40 peptide (30 mg/kg TSA, *p* = 0.000; 60 mg/kg TSA, *p* = 0.001) but also the relatively indissolvable Aβ42 peptide (30 mg/kg TSA, *p* = 0.004; 60 mg/kg TSA, *p* = 0.004), however, the drug efficacy between both doses of TSA had no significant difference. As two primary products, Aβ42 and Aβ40 are hydrolyzed by beta-secretase and gamma-secretase from amyloid-precursor protein. The results implied that TSA perhaps reduced them by inhibiting the production or the activity of secretases participated in the amyloidogenic pathway. Moreover, as indicated in Fig 4C (raw data are presented in S5 Table), the ratio of Aβ42 to Aβ40 increased in the APPswe/PS1dE9 transgenic group (*p* = 0.014), whereas it was unchanged by TSA treatment for 3 months compared with the APPswe/PS1dE9 group (30 mg/kg TSA, *p* = 0.843; 60 mg/kg TSA, *p* = 0.993). To summarize these results, TSA restrained the production of Aβ40 and Aβ42, and the pharmacological function of TSA at a high dose (60 mg/kg) was no better than that of low dose (30 mg/kg).

### Analysis of blood GLUC and plasma CHOL, TG, HDL-C, and LDL-C

As indicated in Table 1, there was no difference in the GLUC [*F* (3, 20) = 0.864, *p* = 0.480] or TG concentrations among the four groups [*F* (3, 20) = 2.221, *p* = 0.125]. The plasma CHOL level was not notably different between the APPswe/PS1dE9 TG group and the WT control group (*p* = 0.968). Treatment with 60 mg/kg TSA decreased the CHOL level compared with the APPswe/PS1dE9 group (*p* = 0.014). We demonstrated that the LDL-C level was increased in the APP/PS1 TG group compared with the WT group (*p* = 0.023). TSA administered by intraperitoneal injection considerably reduced the LDL-C level at both doses relative to the APP/PS1 TG group (*p* = 0.000). The quantity of the HDL-C in the plasma of the APPswe/PS1dE9 transgenic mice was considerably decreased (*p* = 0.001), despite the lack of change in the HDL-C level after treatment with TSA (30 mg/kg TSA, *p* = 0.069; 60 mg/kg TSA, *p* = 0.632).

**Table 1:** Blood GLUC, plasma CHOL, TG, HDL-C and LDL-C of the mice in the four groups (mmol/L).

Group	n	GLUC	CHOL	TG	HDL-C	LDL-C
WT control	5	5.64±0.70	2.82±0.11	0.68±0.06	2.32±0.08	0.42±0.03#
APP/PS1 TG	5	5.22±0.71	2.83±0.28	0.76±0.04	1.62±0.15**	0.55±0.06*
30 mg/kg TSA	5	5.64±0.59	2.33±0.12	0.71±0.10	1.94±0.10**	0.30±0.01*##
60 mg/kg TSA	5	4.48±0.22	2.15±0.14*	1.01±0.16	1.70±0.12**	0.23±0.03**##

CHOL, cholesterol; TG, triglyceride; HDL, high-density lipoprotein; LDL, low-density lipoprotein. Values are in the mean±SEM (*n* = 20).

* *p*<0.05,

** *p*<0.01 versus the WT control group;

^#^*p*<0.05,

^##^*p*<0.01 versus vehicle-treated APPswe/ PS1dE9 transgenic group.

**Fig 5:**
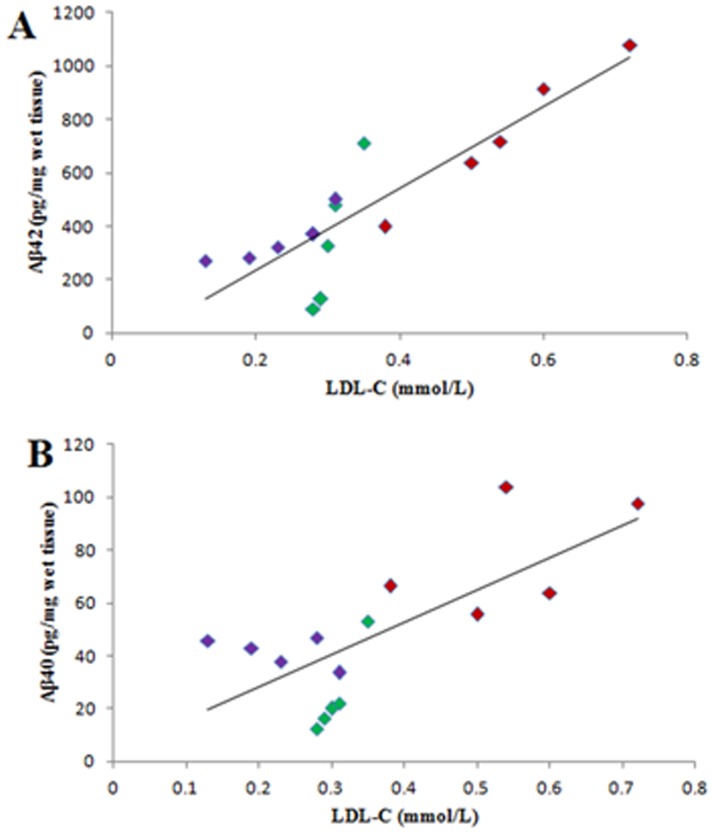
The positive correlations between the levels of the plasma LDL-C and Aβ in the hippocampus of APPswe/PS1dE9 mice were presented in the scatter diagrams. The positive correlation between the levels of plasma LDL-C and the Aβ42 in the hippocampus of 7-month-old APPswe/PS1dE9 mice was illustrated in Fig 5A (*r* = 0.875); the positive correlation between the levels of plasma LDL-C and Aβ40 in the hippocampus of 7-month-old APPswe/PS1dE9 mice was illustrated in Fig 5B (*r* = 0.728). Vehicle-treated APPswe/PS1dE9 transgenic group, red; 30 mg/kg TSA-treated group, green; 60 mg/kg TSA-treated group, purple.

Of note, the plasma LDL-C level had significant positive correlation with Aβ42 level in the hippocampus of 7-month-old APPswe/PS1dE9 mice (*p* = 0.000), and the correlation coefficient (*r*) was 0.875. Similarly, the level of plasma LDL-C was proportional to Aβ40 level in the hippocampus (*p* = 0.002), and the correlation coefficient of them (*r*) was 0.728. The scatter diagrams (Fig 5) suggested that TSA could reduce the levels of Aβ42 and Aβ40 in the hippocampus by decreasing the plasma LDL-C level in 7-month-old APPswe/PS1dE9 mice.

### Multivariate analysis results

**Fig 6:**
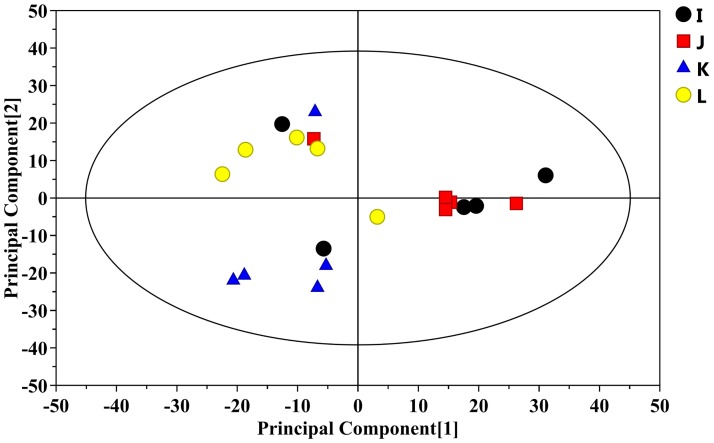
Score plot of the PCA analysis of metabolites for GC-TOF-MS data. I: WT control group; J: vehicle-treated APPswe/PS1dE9 transgenic group; K: 30 mg/kg TSA-treated group; L: 60 mg/kg TSA-treated group. The PCA score plot indicated that the samples were scattered in 4 groups (each sample represented one mouse, *n* = 20). Vehicle-treated APPswe/PS1dE9 transgenic group, red; WT control group, black; 30 mg/kg TSA-treated group, blue; 60 mg/kg TSA-treated group, yellow.

**Fig 7:**
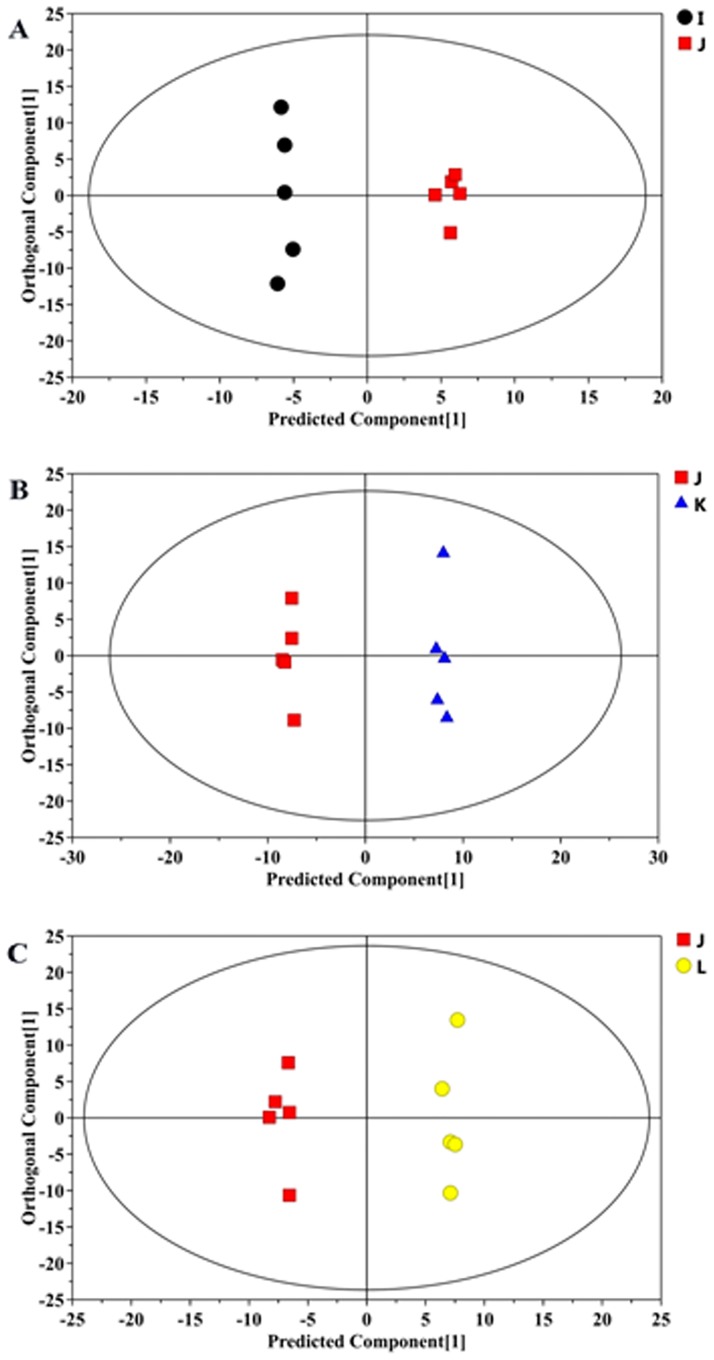
Scores plots from orthogonal signal correction (OPLS-DA) models. Scores plots from OPLS-DA models classifying between the vehicle-treated APPswe/PS1dE9 transgenic group and WT control group (A), the vehicle-treated APPswe/PS1dE9 transgenic group and 30 mg/kg TSA-treated APPswe/PS1dE9 transgenic group (B), and the vehicle-treated APPswe/PS1dE9 transgenic group and 60 mg/kg TSA-treated APPswe/PS1dE9 transgenic group (C). Vehicle-treated APPswe/PS1dE9 transgenic group, red; WT control group, black; 30 mg/kg TSA-treated group, blue; 60 mg/kg TSA-treated group, yellow.

Three hundred fifty-seven peaks were detected, which resulted in 55 metabolites in the denoising method. An unsupervised multivariate statistics-principal components analysis (PCA) initially indicated a modest separation of the four groups; however, it did not separate the four groups absolutely (Fig 6) because of the confounding factor was not eliminated. The metabolite profiles were subsequently detected by OPLS-DA, which comprised a supervised multivariate pattern recognition method. As indicated in Fig 7, the score plots of OPLS-DA described the distribution of the data and displayed a clear distinction between two experimental groups, including the WT control group and APP/PS1 TG group (Fig 7A), as well as the APPswe/PS1dE9 group and two dosed TSA-treated groups (Fig 7B and 7C). R^2^Y and Q^2^Y, the important parameters of the modeling, were 0.993 and 0.438, 0.997 and 0.566, and 0.993 and 0.495, respectively, in the three matched groups, considering that the OPLS-DA method could effectively filter out variant metabolites and adequately explain or predict the variations of the matrices. In addition, the results of two hundred permutations verified the model validity and loading scatter plot of OPLS-DA model was presented between AD and TSA-treated group (original images are presented in S1 and S2 Figs).

### Potential biomarkers

As indicated in Table 2 (raw data are presented in S6 Table), the potential biomarkers of the metabolites were screened according to the OPLS-DA analysis; to further perfect the chemical analysis, VIP values were used to evaluate the contribution of each variable to the model for filtering out the biomarker candidates differentially presented in the vehicle-treated APPswe/PS1dE9 transgenic mice aged 7 months, the 30 mg/kg TSA-treated TG mice and the 60 mg/kg TSA-treated TG mice. Overall, 55 of 357 metabolites were recognized according to the mass spectra and retention indices. Our present research highlighted cholecalciferol (*p* = 0.036) with the greatest potential as a different substance in the hippocampus of the mice in the APPswe/ PS1dE9 group. The substantial decrease in the levels of hippocampus sorbitol (*p* = 0.030) indicated that 7-month-old APPswe/PS1dE9 mice suffered from a latent disorder of sorbitol in the hippocampus. This finding suggested that sorbitol and cholecalciferol may represent candidate biomarkers or the pathogenesis of 7-month aged APPswe/PS1dE9 mice. As a result of autogenous regulation, sorbitol was conclusively increased by 3 months of 30 mg/kg TSA (*p* = 0.003) or 60 mg/kg TSA treatment (*p* = 0.029), and cholecalciferol was decreased by 30 mg/kg TSA (*p* = 0.007) after 3 months of consecutive treatment once per day, which ultimately reversed the impairment of cognitive performance. Glucose-6-phosphate, sucrose-6-phosphate and galactose were not significantly altered (*p* = 0.303; *p* = 0.756; *p* = 0.436) in the APP/PS1 group compared with the control group; however, after 3 months of treatment with 30 mg/kg TSA, glucose-6-phosphate (*p*<0.001) and sucrose-6-phosphate (*p* = 0.008) were substantially increased, whereas galactose was reduced (*p* = 0.008) compared with the APPswe/PS1dE9 transgenic mice. It was noteworthy that although ascorbate was statistically unchanged in the hippocampus of the mice with AD, the *p*-value approached 0.05. A dose of 60 mg/kg TSA increased the level of ascorbate (*p* = 0.018) and decreased the quantity of galactose (*p* = 0.025). The alteration of metabolites after TSA treatment was involved in two types of metabolic pathways, including carbohydrate metabolism, such as sorbitol, glucose-6-phosphate, sucrose-6-phosphate and galactose, and vitamin metabolism, including cholecalciferol and ascorbate. The results indicated that the modulation of these metabolites was helpful to ameliorate the deficits of mice from the aspect of cognition in AD mice.

**Table 2:** Metabolites in the hippocampus of mice identified by GS-TOF-MS as potential markers.

Metabolite	R.T.(min)	Mass (Da)	WT control group *vs*. APP/PS1 group		APP/PS1 *vs*. 30 mg/kg TSA-treated group		APP/PS1 *vs*. 60 mg/kg TSA-treated group	
			Fold change	*p* value	Fold change	*p* value	Fold change	*p* value
sorbitol	17.9217,0	319	2.4921	0.0302#	0.2853	0.0030##	0.3787	0.0291#
cholecalciferol	26.7172,0	357	0.4241	0.0360#	2.0807	0.0070##	2.0973	0.0841
ascorbate	18.0621,0	332	2.8106	0.0598	0.3067	0.1821	0.2204	0.0175#
glucose-6-phosphate	21.3935,0	387	2.4800	0.3030	0.2738	0.0004##	0.3016	0.3320
galactose	17.5859,0	73	0.8013	0.4358	2.9041	0.0079##	2.1689	0.0249#
sucrose-6-phosphate	26.3942,0	217	1.2506	0.7555	0.3605	0.0081##	0.5036	0.1096

Metabolites for the discrimination between WT control and APPswe/PS1dE9 transgenic group, APPswe/PS1dE9 transgenic and 30 mg/kg TSA-treatment group, APPswe/PS1dE9 transgenic and 60 mg/kg TSA-treatment group. Values are in the mean±SEM, *n* = 20.

^#^ *p*<0.05,

^##^ *p*<0.01 versus vehicle-treated APPswe/PS1dE9 transgenic group.

## Discussion

Alzheimer’s disease represents a common type of dementia worldwide. The recent morbidity of AD is greater than ever because of aging and the lack of convincing therapy. Several transgenic mouse lines imitate the age-dependent cognitive impairment of human AD by possessing a high quantity of Aβ in the brains of the mice. AD animal models, such as APPswe/ PS1dE9 mice, accelerate the pathology starting from the age of four months [9]. Traditional cognitive tests associated with the activity of the hippocampus and other brain sections have included the object recognition, MWM and STPA tests, which reflect the neurophysiology of the mice [30]. Based on previous results, cognitive impairments in APP/PS1 mice over 6 months old were observed in the MWM and passive avoidance behavior tasks [31, 32]. The NOR test is a facile behavioral assay with the advantage of little or no stress and needless spatial orientation [33]. A limited number of reports have included the NOR in APP/PS1dE9 transgenic mice in the early period of AD; several papers have illustrated that deficits existed in other transgenic mice related to Aβ at the age of 6–8 months [34], and another study demonstrated that APP/PS1 mice aged 8 months exhibited short-term memory deficits [28]. In our report, the poor exploratory behavior in the NOR elucidated there were deficits in the recognition memory of a short-time in 7-month-old APP/PS1dE9 mice. In this early period of memory impairment, APPswe/PS1dE9 mice were prone to forgetting the object they recently touched. On the other hand, for the APPswe/PS1dE9 mice, perhaps there was less desire to acquaint with the novel object compared with the WT control mice. The next day, the decreased PI of WT control group suggested WT mice could hardly remember the old object they touched 24 hours ago. At that time, no statistical difference of the recognition to the novel object was on account of the shortened gap of PI between APPswe/ PS1dE9 mice and their control mice induced by the slight decline in PI of APPswe/ PS1dE9 mice except for the reduced PI of WT mice 24 hours subsequent to the training. According to such results, a thesis was presented that the impairment of exploratory behavior in the NOR occurred one hour but not 24 hours after the training, which suggested the deficits existed originally in the short-term recognition memory.

On the basis of the second behavior test in our research, we concluded that the unequivocal impairments of spatial learning and memory in the MWM occurred when the APPswe/ PS1dE9 mice were 7 months old, similar to original report [35]published about ten years ago and the conclusion drawn by quite a few specialists in recent manuscripts [36, 37]; however, there was no significant difference in the STPA test, which included a stimulus of electric shock. To date, few reports have investigated preventative TSA-treated APPswe/ PS1dE9 mice concerning behavior tests in the early stage of the disease. In our research of both the NOR and MWM tasks, it was demonstrated that the treatment with TSA reversed the cognitive deficits. It was interesting that only 30 mg/kg TSA ameliorated the impairment of short-term memory in the APPswe/ PS1dE9 mice in the NOR test. It must be further investigated why a high dose of TSA did not have this pharmacological function. During the probe trial in the TSA-treated groups, shorter latencies and increased frequencies across the target platform indicated that the deficits in the spatial memory in the mice with AD were attenuated by TSA treatment.

Vital for spatial and episodic verbal memory, the hippocampus was a pioneer damaged at the earliest stage of AD [38]. APP/PS1dE9 mice exhibited a fast amassing of Aβ inside the hippocampus beginning at approximately the age of 3 months prior to cognitive impairment [39]. As long as TSA had the capability to improve the hippocampus-dependent learning and memory impairments derived from our research, we deduced it would improve in the cognitive function because of the reduced Aβ in the portion of the hippocampus by the preventative treatment of TSA. Thus, we further discussed whether the level of Aβ could be decreased by TSA treatment. A consensus has been reached that the accumulation of Aβ eventually induces tissue injury, neuronal death and cognitive decline [40]. As important constituents of Aβ, two of the most common types are Aβ40 and Aβ42. Aβ40, as the primary peptide of vascular amyloid, composes 90% of the whole secreted amyloid β, and its aggregative speed is relatively slow. As the predominant composition of the SPs [41], Aβ42 is more toxic than Aβ40 in an animal model of AD and may increase the generation of hydrogen peroxide and hydroxyl free radicals, which is the most oxidative stress in vitro [42]. Aβ forms from 3–6 months old in APP/PS1 mice [43, 44]; thus, we conclude the age of 3.5 months in mice may represent a better presymptomatic time point in the initial phase of AD before the plaque and neurodegeneration is severe. In our report, the high concentrations of Aβ42 and Aβ40 and the increased ratio of them in the 7-month-old APP/PS1dE9 mice suggested that a pathologic impairment in the hippocampus of the mice must exist as previously reported [45]. Our results reflected the potential capability of the proportional elimination of the capability of Aβ42 and Aβ40 by 30 mg/kg and 60 mg/kg TSA from the early stage of Aβ formation. It provided preclinical effective evidence of ameliorating the learning and memory impairments of AD as a result of protecting the vascular and neurons from the toxin of Aβ. Regardless of the changes in the behavior of the mice or the deposition of Aβ, the drug efficacy of the high dose of TSA was not better than that of the low dose. In addition, it is necessary to note that Aβ42 and Aβ40 included the total chemical combinations, including the soluble and insoluble formations, because they could solubilize the plaque cores via the means of extracting them [46].

A high-cholesterol diet caused the gathering of amyloid inside the brains of APP mice [47]. It has also been reported that the relation between the LDL-C and Aβ was a positive correlation compared with a negative correlation between the HDL-C and Aβ [48], which is similar to our results. The evidence from our experiment indicated that TSA decreased the Aβ40 and Aβ42 levels by decreasing the plasma LDL-C levels; thus, TSA treatment impacted the lipid metabolism in the plasma, which was helpful to enhance the cognition of the mice with AD. This drug efficacy of TSA has not been reported in previous publications.

TSA treatment affected the substance change in the plasma of mice with AD; thus, we wondered whether it also has an effect on the chemical compound in the hippocampus of mice with AD. Metabolomics using GC-TOF-MS techniques, a beneficial tool for the identification of multiple metabolic alterations, represents the end point of biological reactions during the disease process or therapeutic process of AD [49]. The metabolic profiles inside the hippocampus from the APPswe/PS1dE9 mice and the corresponding TSA-treated mice by GC-TOF-MS demonstrated that several metabolites were identified as potential players in the mice with AD, and the regulation toward these metabolites after TSA treatment was helpful to attenuate the impairments of AD. It was noteworthy that several metabolic perturbations derived from different brain regions in APPswe/PS1dE9 mice have been discussed in a previous report [50]; however, the alterations of other metabolites, such as sorbitol and cholecalciferol, have not been addressed. To our knowledge, the current research comprises the first investigation to identify the characteristic biomarkers of TSA treatment. The utilization of aerobic glycolysis refers to the metabolism from glucose to glucose-6-phosphate and the ultimate product pyruvate from the procedure of which ATP could be rapidly obtained. The regions prone to Aβ deposition were located in the position in which glucose is metabolized via aerobic glycolysis in the brain [51] assessed via PET imaging technology, and the aerobic glycolysis was involved in many glucose-dependent procedures, such as synaptic function and biosynthesis of lipids, nucleic acids and proteins. Using FDG-PET, the structure of FDG is analogous to glucose, and there is a positive correlation between the quantity of FDG in the cell and the metabolism level of glucose by FDG-PET; thus, the progressive hypometabolism indicated by a reduction of FDG in the cerebral metabolite indicated that glucose comprised a vulnerability biomarker of AD involved in nearly all brain regions, which is consistent with the decrease in mental performance [52]. To date, limited studies were reported on the metabolites of aerobic glycolysis in the hippocampus of APPswe/PS1dE9 mice by the method of GC-TOF-MS.

As important energy supplies, glucose and glucose-6-phosphate were reduced in the serum and in the hippocampus of the APPswe/PS1dE9 mice according to previous reports, which may signal a causal factor that leads to AD [50, 53, 54]; however, these levels were not significantly changed in our study in 7-month-old APPswe/PS1dE9 mice. This was thought to be, in part, because of the transformation of part of glucose to sorbitol or the small sample size. It was gratifying that two important energy supply chemical compounds in the hippocampus, such as glucose-6-phosphate and sucrose-6-phosphate, were increased after treatment with 30 mg/kg TSA, whereas the fasting blood glucose level was unchanged. TSA was helpful to convey chemical compounds associated with glucose to the hippocampus passing through the vessels. Glucose-6-phosphate is a vital component that participates in aerobic glycolysis and that cannot pass through the cytomembrance once it enters the cells, and the intracellular accumulation was in favor of glucose absorption. Increasing these productions transformed from glucose represented the increasing activity of synaptic metabolism and the phagotrophic ability of glial cells, which thereby improved mitochondrial dysfunction and neuronal function [55]. These findings suggested that TSA treatment could promote the clearance of Aβ40 and Aβ42 with an increasing energy supply derived from aerobic glycolysis, which is involved in the transformation from glucose to glucose-6-phosphate, the procedure of which has been clinically measured by the 18F-fluorodeoxyglucose (FDG) technique.

The reduced sorbitol converted from glucose in the hippocampus suggested that it lacked essential fuel to help the brain obtain the energy supply in the APPswe/PS1dE9 mice. It also elucidated the influx of glucose encountered an obstacle into the brain across the BBB from the circulation. Sorbitol was transformed from glucose by aldose reductase through the polyol pathway and subsequently oxidized to fructose, which also supported the ATP levels [56]. TSA replenished sorbitol which was the energy supply originated from glucose.

Galactose is a type of monosaccharide classified as an aldose or hexose derived from lactose decomposition. In nature, it exists as D-galactoside in the brain, and a high galactose level may generate ROS and lead to the direct up-regulation of inflammatory cytokines, which thus impairs the astrocyte and cholinergic systems. In addition, galactose may react with amino acids in proteins to produce AGEs-glycoproteins and participate in caspase-mediated apoptosis [57]. These processes cause behavior and cognitive alterations exhibited not only in humans but also in AD animal models; thus, galactose has been used in the induction of aging models [58]. In our study, the data indicated TSA could reduce galactose in hippocampus compared with the APPswe/PS1dE9 mice and thereby resist the production of glycoproteins and other evil effects derived from galactose. TSA ultimately protects the hippocampus from damage and promotes learning and memory.

Cholecalciferol is a neurosteroid hormone with different physiological roles, and it may be produced by nervous system constituent cells [59]. Most previous findings have supported its neuroprotective functions based on its regulation of the expression of neurotrophic factors, calcium homeostasis, pro-inflammatory cytokine secretion and neurotransmission [60, 61]. In the impaired Aβ-rich hippocampus of APPswe/PS1dE9 mice, more endogenesis cholecalciferol was induced and synthesized to resist the destruction by Aβ in our study. TSA sharply decreased the Aβ levels; thus, endogenesis cholecalciferol was reduced after TSA treatment. Nevertheless, as a reactive production, it did not rescue the cognitive decline for the limited protective effect of cholecalciferol.

Several previous experiments have demonstrated that ascorbate comprised a vital neuroprotective substance, and it reduced the impairment of spatial memory in mice with AD [62]. The difference regarding ascorbate in mice and humans is that mice have the ability to synthesize ascorbate in contrast to humans [63]. Previous research has demonstrated the effects of ascorbate on the reversal of memory impairments from the aspects of antioxidant and anti-glutamatergic neurotoxicity properties [64]. Our findings also demonstrated a high dose of TSA induced endogenesis ascorbate synthesis and thus decreased the Aβ levels, which led to cognitive enhancement in learning and memory.

To date, successful disease modifying medicine has met a barrier with the recent failure of AD treatment because of the off-target effects of pharmacology. TSA comprised a type of antioxidative polyphenolic injection extracted from S. miltiorrhiza Bunge, which contained 100 mg of salvia miltiorrhiza polyphenols acid, the main ingredients of which included 60 mg of SalB, 20 mg of SalA and 20 mg of other phenolic acids (SalD, SalE, rosmarinic and alkannic acid). As a traditional herbal medicine, it has been utilized as a therapy to treat cerebrovascular diseases, such as cerebral ischemia etc [65]. In recent years, novel drug efficacies of Salvia have been disclosed, such as improvements in microcirculation, anti-platelet-aggregation and thrombosis [66]. Nevertheless, the research on AD treated with TSA remains limited to date. In vitro, SalB restrains the gathering of Aβ, fibril formation and cellular toxicity derived from amyloid [67]. Previous in vivo studies have been confined to the investigation of animal models of AD by short-term Aβ injection, which cannot mimic the gradual pathological changes of AD; moreover, despite evidence that salvianolic acid had neuroprotection via antioxidative effects, it has not been elucidate whether salvianolic acid could resist the impairment of long-term Aβ accumulation [22, 67]and reason of drug effectiveness from the aspect of metabonomics. The study was a tentative exploration on the pharmacology of TSA which was effective to treat APP/PS1 mice comprised AD animal model about metabonomics seldom reported previously.

## Conclusion

In our study, we concluded that TSA comprised a multi-metabolite regulator whose pathway was involved in carbohydrate metabolism and vitamin metabolism in the hippocampus and lipid metabolism in plasma, which could be used in the earlier stages of AD prior to severe Aβ deposition and neurodegeneration. To our knowledge, this investigation comprises the first report that demonstrated TSA may represent a promising therapeutic candidate for AD because of its sustainable protective effects on learning and memory via metabolite regulation, as demonstrated in this study. In the future, second validation detection should be conducted using a targeted approach to confirm the current findings; moreover, the mechanisms of the metabolite alterations remain to be elucidated. In addition, the plasma metabolites in the mice should be investigated. The disadvantage of our study including short of pathological research will be subsequently addressed in more detail.

## Supporting information

S1 Fig Permutation test between APP/PS1 and 30 mg/kg TSA-treated group. Two hundred permutations were performed, and the resulting R^2^ and Q^2^ values were plotted. Green circle: R^2^; blue square: Q^2^. The green line represents the regression line for R^2^ and the blue line for Q^2^. The intercepts of R^2^ and Q^2^ in permutation test were 0.924 and -0.0503. The results suggested the robustness of the model.
(TIF)

S2 Fig Loading plot of potential biomarker in OPLS-DA model between APP/PS1 and 30 mg/kg TSA-treated group. (TIF)

S1 Table Preference index of the four groups in the NOR experiment. (PDF)

S2 Table Effects of TSA on escape latency of 7-month-old APP/PS1 mice in the spatial navigation. (PDF)

S3 Table Effects of TSA on memory of 7-month-old APP/PS1 mice in spatial probe trial. (PDF)

S4 Table Latency of mice in step-through passive avoidance task. (PDF)

S5 Table Aβ42 and Aβ40 levels and the ratio of them in the hippocampal homogenates of mice. (PDF)

S6 Table The corresponding normalized peak area of metabolites in the hippocampus of mice identified by GS-TOF-MS. (PDF)

## Abbreviations

Aβ: amyloid β-peptide
AD: Alzheimer’ s disease
AGEs: advanced glycation endproducts
BSAT-DPBS: Dulbecco’s phosphate buffered saline with 5% BSA and 0.03% Tween-20
CHOL: cholesterol
FBG: fasting blood glucose
GC-TOF-MS: gas chromatography coupled to time-of-flight mass spectrometry
GOPOD: glucose oxidase and peroxydase
HDL: high-density lipoprotein
LDL: low-density lipoprotein
MSA: multivariate statistical analysis
MWM: Morris water maze
NFs: intracellular neurofibrillary tangles
NOR: novel object recognition
PI: preference index
ROS: reactive oxygen species
SPs: senile plaques
TG: triglyceride
TSA: total salvianolic acid
VIP: variable importance projection
