# Supplementary figures and images for: Amelioration of cognitive impairments in APPswe/PS1dE9 mice is associated with metabolites alteration induced by total salvianolic acid
Source: PLoS One. 2017 Mar 30;12(3):e0174763. doi: 10.1371/journal.pone.0174763 (PMC5373599; doi:10.1371/journal.pone.0174763)

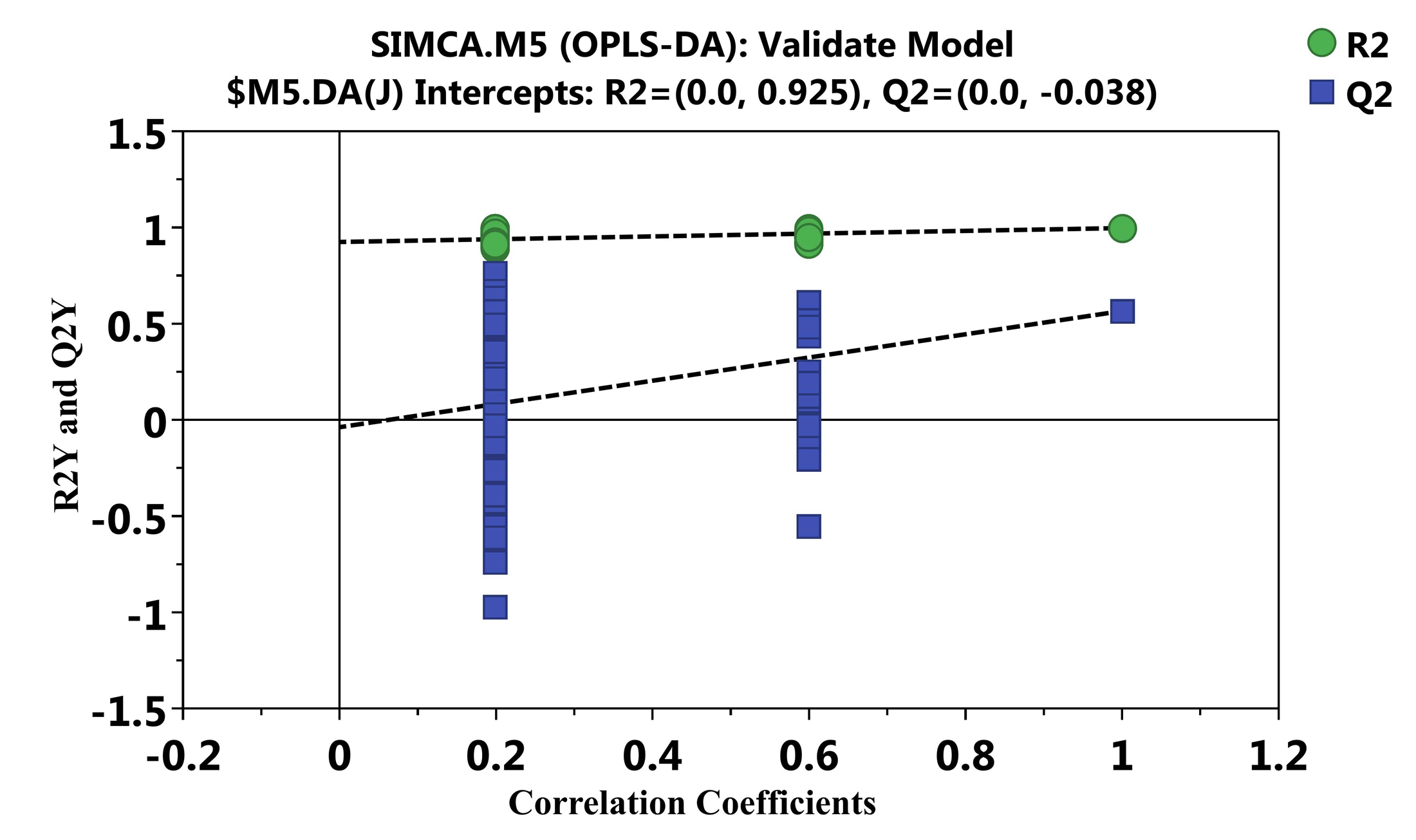

Supplement: S1 Fig — Two hundred permutations were performed, and the resulting R2 and Q2 values were plotted. Green circle: R2; blue square: Q2. The green line represents the regression line for R2 and the blue line for Q2. The intercepts of R2 and Q2 in permutation test were 0.924 and -0.0503. The results suggested the robustness of the model. (TIF) [file pone.0174763.s001.tif]

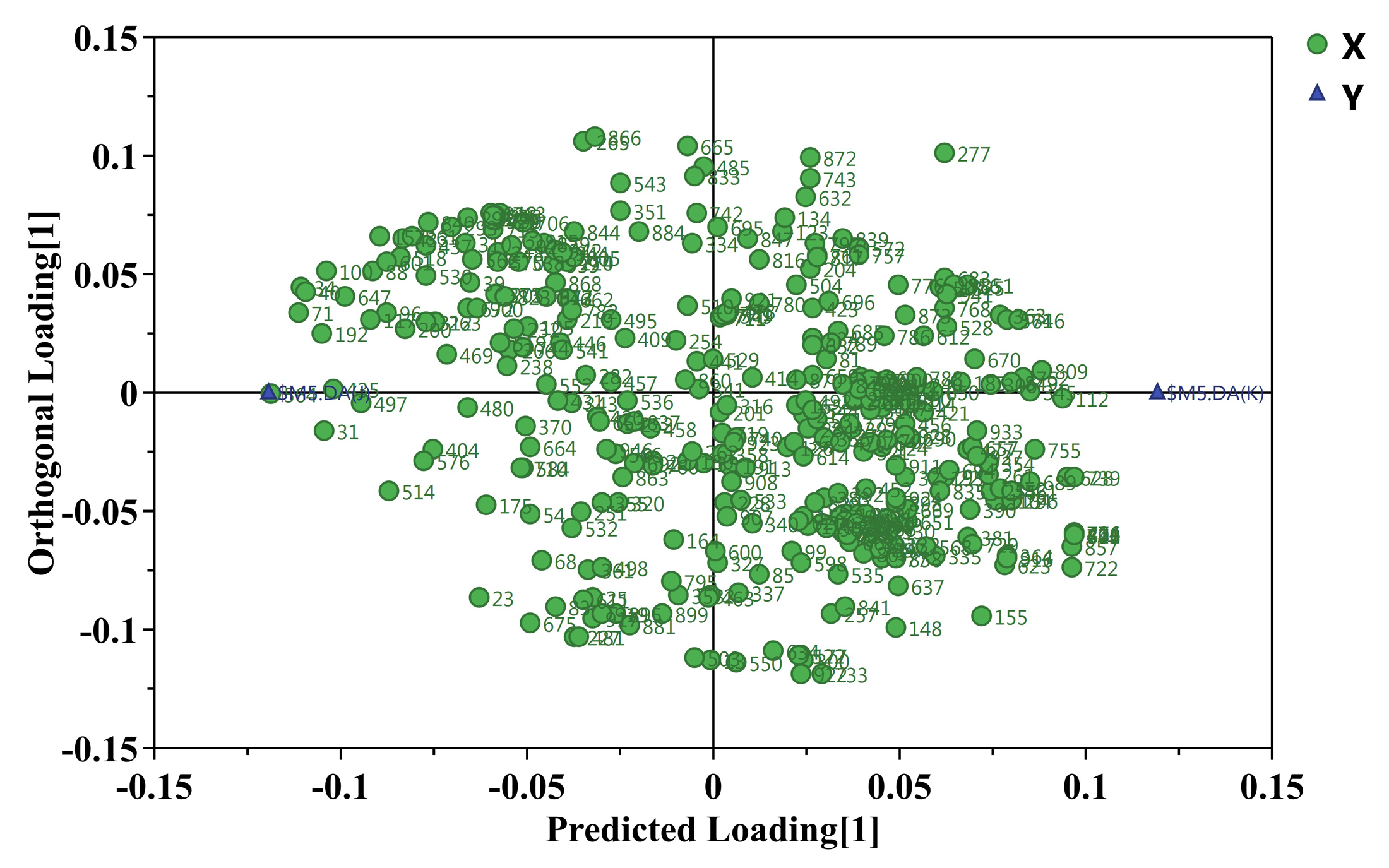

Supplement: S2 Fig — (TIF) [file pone.0174763.s002.tif]
