# Supplementary material for: Amelioration of cognitive impairments in APPswe/PS1dE9 mice is associated with metabolites alteration induced by total salvianolic acid
Source: PLoS One. 2017 Mar 30;12(3):e0174763. doi: 10.1371/journal.pone.0174763 (PMC5373599; doi:10.1371/journal.pone.0174763)
Supplement: S1 Table — (PDF) [file pone.0174763.s003.pdf]

S1 Table Preference index of the four groups in the NOR experiment.

Group	n	1H	24H
		mean ±SE	mean ±SE
WT control	5	0.848 ±0.068##	0.739 ±0.110
APP/PS1 TG	5	0.584 ±0.055**	0.655 ±0.024
30 mg/kg TSA	5	0.933 ±0.049##	0.798 ±0.131
60 mg/kg TSA	5	0.644 ±0.040*	0.602 ±0.059

Note: *p<0.05, **p<0.01 *vs* WT control group; #p<0.05, ##p< 0.01 *vs* APP/PS1 transgenic group.
