# Supplementary material for: Amelioration of cognitive impairments in APPswe/PS1dE9 mice is associated with metabolites alteration induced by total salvianolic acid
Source: PLoS One. 2017 Mar 30;12(3):e0174763. doi: 10.1371/journal.pone.0174763 (PMC5373599; doi:10.1371/journal.pone.0174763)
Supplement: S2 Table — (PDF) [file pone.0174763.s004.pdf]

S2 Table Effects of TSA on escape latency of APP/PS1 mice in the spatial navigation trials (mean ±SE).

Group	n	Escape latency				
		1st day	2nd day	3rd day	4th day	5th day
WT control	5	45.294 ±6.876	53.406 ±4.100	41.140 ±6.409	23.568 ±5.102	23.810 ±6.296##
APP/PS1 TG	5	50.009 ±6.701	49.170 ±3.795	48.128 ±4.918	41.064 ±7.125	43.676 ±4.453**
30 mg/kg TSA	5	45.984 ±4.266	49.398 ±4.817	27.712 ±3.520	26.240 ±4.902	23.780 ±4.349##
60 mg/kg TSA	5	57.090 ±2.151	47.110 ±4.710	34.697 ±7.006	33.382 ±6.076	27.542 ±2.263#

Note: *p<0.05, **p<0.01 *vs* WT control group; #p<0.05, ##p< 0.01 *vs* APP/PS1 transgenic group.
