# Supplementary material for: Amelioration of cognitive impairments in APPswe/PS1dE9 mice is associated with metabolites alteration induced by total salvianolic acid
Source: PLoS One. 2017 Mar 30;12(3):e0174763. doi: 10.1371/journal.pone.0174763 (PMC5373599; doi:10.1371/journal.pone.0174763)
Supplement: S3 Table — (PDF) [file pone.0174763.s005.pdf]

S3 Table Effects of TSA on memory of 7-month-old APP/PS1 mice in spatial probe trials (mean±SE).

Group	n	Latency (s)	Frequency of platform crossings	Time ratio of target quadrant (%)
WT control	5	21.440±1.224##	1.800±0.583#	22.880±2.121#
APP/PS1 TG	5	56.372±3.491**	0.400±0.245*	13.300±3.663*
30 mg/kg TSA	5	18.282±4.089##	3.000±0.894#	17.454±0.780
60 mg/kg TSA	5	19.082±3.718##	2.000±0.775#	21.718±2.256#

Note: *p<0.05, **p<0.01 *vs* WT control group; #p<0.05, ##p< 0.01 *vs* APP/PS1 transgenic group.
