# Supplementary material for: Amelioration of cognitive impairments in APPswe/PS1dE9 mice is associated with metabolites alteration induced by total salvianolic acid
Source: PLoS One. 2017 Mar 30;12(3):e0174763. doi: 10.1371/journal.pone.0174763 (PMC5373599; doi:10.1371/journal.pone.0174763)
Supplement: S4 Table — (PDF) [file pone.0174763.s006.pdf]

S4 Table Latency of mice in step-through passive avoidance task (s).

Group	n	Step-through latency
WT control	5	161.240±9.086
APP/PS1 TG	5	66.952±35.100
30 mg/kg TSA	5	175.808±4.456
60 mg/kg TSA	5	104.938±34.530

Note: Data are presented as the mean±SEM, *n*=20.
