# Supplementary material for: Amelioration of cognitive impairments in APPswe/PS1dE9 mice is associated with metabolites alteration induced by total salvianolic acid
Source: PLoS One. 2017 Mar 30;12(3):e0174763. doi: 10.1371/journal.pone.0174763 (PMC5373599; doi:10.1371/journal.pone.0174763)
Supplement: S5 Table — (PDF) [file pone.0174763.s007.pdf]

S5 Table Aβ42 and Aβ40 levels and the ratio of them in the hippocampal homogenates of mice (mean ±SE).

Group	n	Aβ42 (pg/mg)	Aβ40 (pg/mg)	Aβ42/Aβ40
WT control	5	1.304 ±0.143##	8.051 ±0.361##	0.162 ±0.016#
APP/PS1 TG	5	749.103 ±117.136**	77.496 ±9.741**	9.942 ±1.544*
30 mg/kg TSA	5	346.505 ±114.879*##	24.772 ±7.287*##	13.296 ±2.692*
60 mg/kg TSA	5	348.135 ±41.575*##	41.378 ±2.515**##	8.743 ±1.605*

Note: **p*<0.05, ***p*<0.01 versus WT control group; #*p*<0.05, ##*p*<0.01 versus vehicle-treated APPswe/PS1dE9 transgenic group.
