# Supplementary material for: Amelioration of cognitive impairments in APPswe/PS1dE9 mice is associated with metabolites alteration induced by total salvianolic acid
Source: PLoS One. 2017 Mar 30;12(3):e0174763. doi: 10.1371/journal.pone.0174763 (PMC5373599; doi:10.1371/journal.pone.0174763)
Supplement: S6 Table — (PDF) [file pone.0174763.s008.pdf]

S6 Table The corresponding normalized peak area of metabolites in the hippocampus of mice identified by GS-TOF-MS.

Metabolite	WT control ($\times 10^{-6}$)	APP/PS1 TG ($\times 10^{-6}$)	30 mg/kg TSA ($\times 10^{-6}$)	60 mg/kg TSA ($\times 10^{-6}$)
sorbitol	341.06±52.25#	136.86±57.43	479.72±57.77##	361.41±62.13#
cholecalciferol	609.46±306.60#	1436.96±118.92	690.61±169.86##	685.14±362.19
ascorbate	290.78±32.10	103.46±79.26	337.35±139.07	469.49±93.58#
glucose-6-phosphate	247.88±123.94	99.95±26.75	364.99±36.74##	331.40±209.05
galactose	27941.38±5910.77	34871.40±6038.52	12007.54±2433.38##	16077.82±3175.81#
sucrose-6-phosphate	80.70±39.70	64.53±30.66	179.02±11.44##	128.16±17.58

Note: Values are in the mean±SEM, n=20. #p<0.05, ##p< 0.01 *vs* APP/PS1 transgenic group.
